# Shifting palliative care paradigm in primary care from better death to better end-of-life: a Swiss pilot study

**DOI:** 10.1186/s12913-021-06664-1

**Published:** 2021-07-01

**Authors:** Johanna Sommer, Christopher Chung, Dagmar M. Haller, Sophie Pautex

**Affiliations:** 1 Faculty of Medicine Geneva, University Institute of Primary Care, University of Geneva, Centre Médical Universitaire, Rue Michel-Servet 1, 1211, Genève 4, Switzerland; 2 University Hospital of Geneva, Geneva, Switzerland

**Keywords:** Cancer care / oncology, Death and dying, Doctor-patient relationship, Palliative care / end-of-life, Primary care, Quality of life

## Abstract

**Background:**

Patients suffering from advanced cancer often loose contact with their primary care physician (PCP) during oncologic treatment and palliative care is introduced very late.

The aim of this pilot study was to test the feasibility and procedures for a randomized trial of an intervention to teach PCPs a palliative care approach and communication skills to improve advanced cancer patients’ quality of life.

**Methods:**

Observational pilot study in 5 steps. 1) Recruitment of PCPs. 2) Intervention: training on palliative care competencies and communication skills addressing end-of-life issues. 3) Recruitment of advanced cancer patients by PCPs. 4) Patients follow-up by PCPs, and assessment of their quality of life by a research assistant 5) Feedback from PCPs using a semi-structured focus group and three individual interviews with qualitative deductive theme analysis.

**Results:**

Eight PCPs were trained. Patient recruitment was a challenge for PCPs who feared to impose additional loads on their patients. PCPs became more conscious of their role and responsibility during oncologic treatments and felt empowered to take a more active role picking up patient’s cues and addressing advance directives. They developed interprofessional collaborations for advance care planning. Overall, they discovered the role to help patients to make decisions for a better end-of-life.

**Conclusions:**

While the intervention was acceptable to PCPs, recruitment was a challenge and a follow up trial was not deemed feasible using the current design but PCPs reported a change in paradigm about palliative care. They moved from a focus on helping patients to die better, to a new role helping patients to define the conditions for a better end-of-life.

**Trial registration:**

The ethics committee of the canton of Geneva approved the study (2018–00077 Pilot Study) in accordance with the Declaration of Helsinki.

## Background

Cancer is one of the main causes of death in Switzerland. Each year nearly 17′000 people will die with cancer as main diagnosis (29,4% of all causes of mortality for men and 22,5% for women [1]. Primary care physicians (PCPs) mostly deliver the diagnosis, then patients are referred to oncologists for management and treatment planning [2]. During this period there is often a loss of contact between patients and PCPs. When cancer becomes life-limiting and unlikely to be cured, oncological treatments may no longer be appropriate and a transition back to the PCP usually occurs [2].

However, this transition often occurs very late. A palliative care approach is introduced in the last weeks or days of life of patients, advanced directives are poorly defined, and psychological and spiritual needs are minimally supported [3, 4]. This suggests PCPs could be involved much earlier to assess and manage the multidimensional needs of these patients. These needs include the management of pre-existing comorbidities, coordination of care between the different healthcare providers, management of treatments’ side effects and symptoms due to cancer [5–7]. PCPs need to regularly reassess patients’ needs and to know their patients’ wishes and preferences for end-of-life care. Furthermore, patients’ spiritual needs have to be recognised and considered [8–10].

A systematic review identified barriers to the introduction of this palliative care approach: lack of time, lack of training, ambivalence towards discussing end-of-life issues and lack of communication skills [11]. Swiss PCPs also expressed uncertainty and discomfort communicating about these subjects with patients [12]. This highlighted a need for PCPs to access improved training in palliative care approaches and communication with advanced cancer patients and to receive support in end of life care [11, 13]. PCPs need good communication skills for complex conversations with intense social, psychological and spiritual significance [6, 10, 11, 14–18]. They should feel comfortable to talk about end-of-life issues, as well as important values or personal goals for care, preferences for types of care, and preferred place of care or dying. They need to know how to initiate a shared decision-making process and advance care planning [19]. Yet to date we lack evidence in relation to the effectiveness of training PCPs in palliative care on the quality of patients’ end-of-life care.

The purpose of the present study was to assess the feasibility of an educational intervention for addressing end-of-life issues tailored for PCPs and to identify best strategies and barriers for the recruitment of outpatients with advanced cancer. The final aim of this study was to develop a randomised trial to test the effectiveness of a PCP tailored intervention to improve outpatient’s quality of life with advanced cancer.

## Methods

Our objectives relate to the assessment of the feasibility of the intervention, the proposed recruitment strategies and outcome assessment protocols:

1. Determine best strategies as well as recruitment and retention rates of PCPs.
2. Determine the acceptability of the intervention for the PCPs (engagement and compliance with the intervention).
3. Identify best strategies for recruitment and retention of the patients with advanced cancer by their PCPs.
4. Test procedures and outcome measurements for this randomised study to assess the effectiveness of a PCP tailored intervention.

### Study design

The pilot study was divided in five parts:

- Recruitment of PCPs
- Training of PCPs with the tailored intervention
- Recruitment of the patients by the PCPs
- Patient follow-up by the PCPs and assessment of their quality of life by a research assistant
- Feedback from PCPs in relation to the training and the study procedures

### Recruitment of the PCPs

We recruited PCPs among a convenience sample of primary care medical teachers attached to our university in the French speaking part of Switzerland. Fifty emails were sent in April 2018 to invite PCPs to participate to this study. We planned that approximately 10 PCP would agree to participate.

### The training of the PCPs

To participate, all PCPs had to attend two half-day training sessions between June and August 2018: outline Table 1). The aim of these sessions was to facilitate PCPs’ active role in keeping contact with advanced cancer patients, daring to address with the patient a so-called “plan B” (the treatment plan if the oncological treatment is no longer effective, or if the patient no longer tolerates it). The training sessions covered the themes that usually need to be addressed with advanced cancer patients: advance directives; communication about early palliative care, patient care preferences, and advance care planning; palliative care network presented by two nurses; and palliative care clinical care strategies (symptoms and pain assessment, pain treatment strategies, screening for severe symptoms) (Table 2). The workshops were delivered jointly by a palliative medicine consultant (SP); a senior PCP with expertise in palliative care and in postgraduate communication education for the role plays (JS); a junior PCP trained in research (CC); two palliative care nurses; and two trained simulated patients.

**Table 1:** Outline of the two half days training

1st half-day	Title	Goal, learning objective	Method
1:30 pm	Introduction	Presentations, program	Oral exchange
1:40 pm	What challenges when caring for severe cancer patient as GP?	Ice breaking, expectations	Reflection in pairs, sharing in plenary
1:50 pm	Witness of a patient’s husband expressing experience of palliative care for his wife at home	catch attention	Video
2:00 pm	What is a “good” death?	Clarification of expectations and fears	Reflexion in pairs, sharing in plenary
2:30 pm	Project presentation	Presentation of project steps, inclusion criteria, material (questionnaires, etc), network and resources	Power point and discussion
	How to explain the project to my patient?	Training of recruitment process	Role play in pairs
	Questions-Answers	Clarify project	Answer questions
3:00 pm	*Break*		
3:30 pm	Symptoms assessment	Assess frequent symptoms in palliative care	Power point
		Use of Edmonton symptom assessment scale	Role play in pairs
4:00 pm	Treatment of pain	Review strategies for treatment	Power point
4:15 pm	Existing local network in palliative care	Presentation of existing network by teams (nurses and doctors)	Oral presentation and power-point, document
4:30 pm	Advance directives (AD)	Presentation of legal and practical background	Powerpoint
		Practice discussion on AD	Role play with simulated patients in two groups
5:25 pm	Conclusions	Share on what was learned	Plenary
**2nd Half day**			
1:30 pm	Introduction	Sharing experiences	Plenary
2:00 pm	How to deal with palliative care emergencies (dyspnea, nausea, neurologic symptoms, etc	Recognize and dealing with palliative care emergencies	Power point and discussion based on participants’ experiences
3:00 pm	*Break*		
3:30 pm	How to plan advance care	Presentation of intermediate consultations possible topics (patient preferences and values, choice of end-of-life care and place, spiritual and psychological needs	Power point and discussion in plenary
3:50 pm	How to address difficult conversations	Discover and experience “go-wish” cards	Practice in pairs (and with go-wish cards)
4:10 pm	How to address difficult conversations?	Practice difficult conversations	Role play with simulated patients in 2 groups
5:15 pm	How to say good-bye?	Reflection on difficult separations with patients	
		Reflection on key messages and practice how to say goodbye	Exercise in pairs
5: 25 pm	Conclusions	Reflection on key messages	Plenary

**Table 2:** Possible important themes to address with advanced cancer patients

• Advance care planning (including emergency plan if worsening health status during nights or week-ends)	
• Gravity of illness, fears, patient’s representations	
• Patient’s needs, wishes, important life aims, projects to achieve in coming time	
• Psychosocial needs	
• Spiritual needs	
• Relative’s needs	
• Financial aspects, worries	
• Wished place of care and of end-of-life	

Effective ways of discussing end-of-life issues with patients were explored through an interactive role-play with simulated patients.

At the end of the training, participants completed a questionnaire assessing their perspective on the organisation; content; teaching methods; quality of teachers; extent to which the training met their expectations and global satisfaction, using a 5-point Likert scale. They also completed 4 open questions asking them about lessons learned; suggestions for improvement, unmet needs and overall satisfaction).

### Recruitment of advanced cancer patients by the PCPs

We planned that each PCP would identify 1–3 patients.

Inclusion criteria were: Patients with advanced cancer followed by the PCP; fluent in French.

Exclusion criteria: imminent death. The recruitment period was from September 2018 to December 2019.

### Follow-up of patients by the PCPs

It consisted in four consultations with the PCP that were paid by the study over 6 months. The aim of these consultations was to keep contact between the PCP and the patient during the oncologic treatment and to address any useful content described in Table 2. PCP’s didn’t have to notify patients’ consultations.

Patients were asked to complete a questionnaire via phone by a research assistant (MM). In addition to patients’ personal data, the following data were collected (at time 0, + 3 months, + 6 months): symptom scales (Edmonton symptom assessment [20], quality of life scale (SF12v2) [21], anxiety and depression scale (hospital anxiety and depression scale) [22], spiritual and care values (FACIT SP) [23]).

### PCPs’ feedback in relation to the training and the study procedures

Focus groups were planned with the PCPs to collect their feedback concerning the intervention, the recruitment and the follow-up consultations of the patients and the strategies and barriers to participation in a randomised study. The method had to be adapted online because of the COVID-19 pandemic. The interviews were conducted by JS (MD), known by participants as PCP, colleague and clinical teacher at the university. A content analysis was done qualitatively by inductive theme analysis on an audio-recording of the session partly verbatim retranscribed (relistened more than 3 times but the introductions and small talk were not transcribed); themes were comparatively analysed by the two authors (JS and SP) until full agreement on themes. The Table 3 (Table 3: Guide for semi structured focus -group /interview) details the semi-structured interview guide (pilot-tested among clinical teachers of the teaching unit).

**Table 3:** Guide for semi structured focus -group /interview

1) What lessons were learned from the project?	
2) What changed in your practice?	
3) What changed regarding the advanced directives (completed protocolled form)?	
4) What changed regarding an advanced care planning?	
5) What changed regarding your integration of palliative care into your practice?	
6) What changed in your way to integrate the family into the care of advanced cancer patients?	
7) What problems did you face during the project?	
8) What would you suggest as improvement for a next study?	

The primary care clinicians gave informed consent to group or individual interviews after the intervention, and also gave informed consent to participate in the workshops and the study, as well as for publication of results.

The patients provided a signedinformed consent to participating in the study. The study was approved by the ethics committee of the canton of Geneva in accordance with the Declaration of Helsinki (2018–00077 Pilot Study: continuous follow-up by primary care physicians facilitating early palliative care for patients with advanced cancer disease).

## Results (Fig. 1: flow chart of pilot study)

### Recruitment of the PCPs

Ten PCPs were willing to participate to the study but two could not participate to the two half day courses, so we included 8 participants (Description of participating PCPs: Table 4). Two participants were relatively experienced in palliative care (GP2 and GP5). Interestingly the PCP who had the most recent experience in palliative care (> 20 patients in last year) did not have any previous training, and only one PCP had more than 10 h previous training in palliative care.

**Fig. 1:**
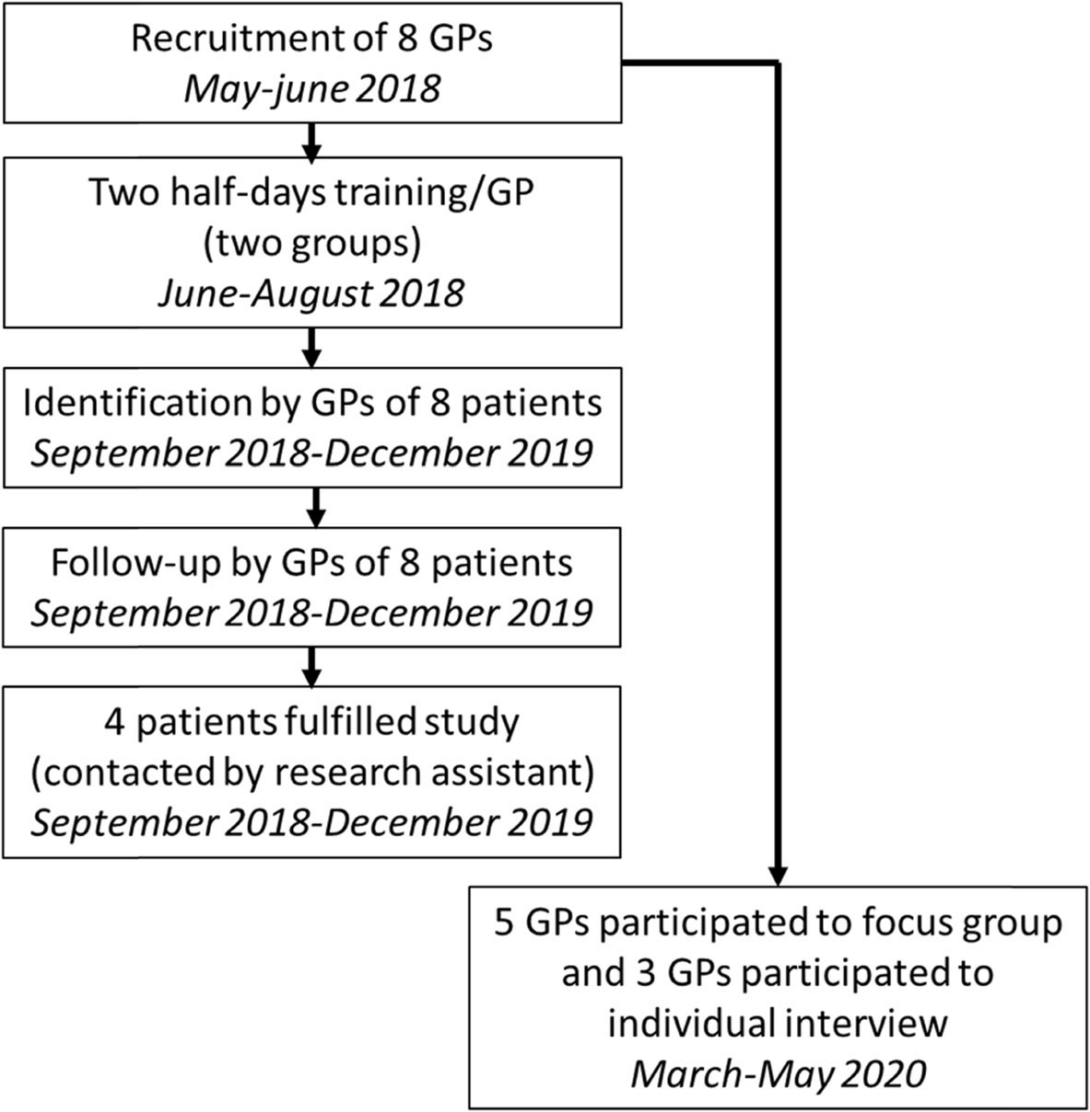
Synopsis of pilot study

**Table 4:** Description of participating PCPs

	Sex	Age (range in years)	Years in practice	Home visits per week	Previous course in palliative care	Hours training in palliative care	N of palliative care patients in last year as attending physician	Sensibilized patients for study	Participation to focus group (FG) or individual interview (II)
GP1	F	55–59	12	5	Yes	> 5	2	0	FG
GP2	M	50–54	12	7	Yes	> 5	12	2	II
GP3	M	50–54	14	4	No	none	2	1	FG
GP4	F	45–49	4	2	Yes	> 10	0	2	II
GP5	M	45–49 years	10	10	No	none	20	1	FG
GP6	F	50–54 years	13	10	Yes	> 5	0	0	FG
GP7	F	50–54	13	2	No	none	0	2	FG
GP8	F	45–49	10	2	No	none	0	0	II

### Training of PCPs

Two sessions were organised (1st session 5th June and 7th June 2018; second session 19th June and 28th August 2018). All PCPs participated in both workshops.

Only half the participants completed the evaluation form: they reported a mean 10/10 global satisfaction with the training.

### Recruitment of advanced cancer patients by the PCPs

Though we sent five emails to remind PCPs to recruit patients, eight patients only were recruited by four PCPs and only four patients could finally be included.

### Patients’ follow-up

Only one patient completed the baseline and two follow-up questionnaires, two patients completed both the baseline and first follow-up questionnaire whereas one patient only completed the baseline questions.

Two other patients could never be joined by phone, and two declined participation at the first phone call.

### PCPs’ feedback in relation to the training and the study procedures

Five out of eight PCPs participated in the focus group (90 min) by zoom in April 2020 (we avoided contact because of COVID-19 pandemic), and the other three answered individually the same questions during a semi-structured interview by phone (30 min).

The thematic inductive analysis revealed five different themes, data saturation was achieved after the focus group and the first individual interview.

#### Qualitative analysis of focus groups

##### PCPs’ role

PCPs felt empowered and legitimated to take a more active role, and to keep contact with their patients during oncologic treatments.

*“It is now clear for me that it is important to walk together on the same path with the patient and the oncologist so as to avoid this parallel path on which we feel losing contact with our cancer patients...”GP2*

They expressed being more conscious of their role and responsibility and more comfortable to discuss treatments with their advanced cancer patients, as well as to address their preferences, and anticipate for worsening situations.

##### Changes in practice

Participants reported being more active to contact patients by phone during their oncologic treatment.

*“I proactively called patients where I had lost contact (during their oncologic treatment), and I felt it legitimate ... …I felt before like embarrassed…GP3*

*Yes, me too, and every time I called, patients were amazed… positively… and it ended on very important discussions” GP7*

The interprofessional collaboration for advance care planning became evident, aiming the best care for the patient.

*“I developed the thought of anticipation together with an interprofessional team, defining the advance care plan, and sharing it in a written form with all other concerned healthcare providers… this completely changed my relationship with my network, because everyone was reassured, to know everyone’s role, and knowing what to do next, this changed a lot in situations where sometimes everyone felt exhausted” GP7*

Palliative care has changed its significance and definition, from what was considered end-of-life care, into a larger view of aiming for the best treatment for the patient and anticipating negative health issues. This means foreseeing the actions in case of worsening, for instance what can be done without hospitalisation and aggressive treatments, as well as adjusting care to patient’s preferences.

*“I changed my definition of palliative care…it was the treatment for the 2-3 last days of life…I now try, with the interprofessional team, to adapt better to patient preferences of care”.*

*“It is not an alternative anymore between curative and palliative, but series of options… to find out with the patient “GP7*

It was reported that PCP learned to have a broader vision of patient’s needs and to screen for other subjective symptoms than just pain (for instance anxiety, loss of appetite, depression).

*“It helped me to better support the emotions, and I feel better equipped to support patients when having emotional discomfort or other symptoms, ...and I feel less helpless”. GP6*

PCPs expressed feeling more legitimate as the patient’s advocate in front of specialists or hospital caregivers, being conscious of knowing the patient well especially if advance care planning discussions had taken place.

##### About advance directives and advance care planning

Participants reported thinking more actively to define advance directives and to pick up cues from patients more easily and earlier to address the subject.

*“I realized that it is easier to speak about advance directives when patients are not feeling too bad, so I address the subject much earlier” GP3*

The paradigm about advance directives has changed becoming a more positive role for the PCP to help patients to make decisions for a better life.

*“Before I thought that advance directives were the way of dying and felt uncomfortable addressing this subject, but now it is more helping a patient to decide personally for the choices of care and for his wishes for continuing life” GP1*

##### Difficulties recruiting patients

PCPs found it difficult to recruit patients for fear of imposing too important a load. They did not feel comfortable to let a person who is not involved in patients’ care (research assistant) “annoying” patients with the questionnaires.

Though they follow patients with metastatic cancer, they expressed it was difficult to consider them as “advance cancer patients”, like if they were condemning patients if they recruited them into the study.

*“though we try to increase quality of life, we take the risk to take the hope away, when we speak about palliative care” GP1*

*“It’s difficult for us, ...maybe unconsciously we don’t accept that it’s the end. So it’s hard to talk about it, we would like to give hope” GP2*

They reported some patients not wanting to address end-of-life issues, making it impossible to recruit them.

*“One of my patients refused to address the subject of possible negative issue” GP1*

PCPs expressed the difficulty to use the words “palliative care” with patients, because associated to death, and suggested to rename it: “*support treatment*” or, “*patient support*”. Some PCPs found it very important to clarify the palliative attitude with the patients and families.

## Discussion

This pilot study explored the feasibility of a study improving follow-up by PCPs of advanced cancer patients, including early palliative care and involvement in the development of treatment care plan and advance directives.

This feasibility study showed that recruitment was not be feasible for a RCT using the current design as there were very few patients recruited and even fewer who completed the follow-up questionnaires. Asking palliative care patients to complete such questionnaires is probably too demanding and many patients secondarily declined to complete the questionnaires..

We aimed to recruit 10 PCPs: 50 emails were sent as invitation and 8/50 doctors participated. Recruiting PCPs for research is known to be difficult [24] and this rate did not surprise us.

However, the two half-day workshops were much appreciated and had a positive effect on PCPs’ attitudes and actions towards their advanced cancer patients. All PCPs expressed great satisfaction with the intervention showing great acceptability of the intervention. The qualitative analysis of the focus groups showed that PCPs felt empowered to take an active role in the follow-up of their patients during oncologic treatment. As described in the literature, PCPs felt responsible to initiate end-of-life discussions, arising from their role and expertise, and these conversations’ benefits [25, 26]. PCPs felt better equipped in communication skills, and picked up cues more easily to address advance directives or end-of-life discussions, having practiced in role plays and made their own experience with patients. Training and coaching of non-palliative care clinicians such as PCPs can increase the practice of palliative care for the patients. Trained PCPs identify more palliative patients than do untrained PCPs and the non-palliative care clinicians report increased comfort and skill at conducting goals of care conversations [27].

For patients and PCPs alike, palliative care has a negative image as it is related to death [28]. In the literature, PCPs’ barriers to communicating on these subjects were described as: concerns about patients’ readiness for end-of-life discussions or their potential psychological impact [18]; family conflict [29]; cultural factors [30–32]; prognostic uncertainty [15, 33]; and inexperience [25, 29]. This prevents PCPs from addressing these difficult subjects, and in our study, probably contributed to their difficulties recruiting patients. Very interestingly, the definition of palliative care changed during the study for the participating PCPs: they reported investing their role as facilitator for a better definition of patients’ care preferences instead of having to address issues related to death. They felt able to avoid unnecessary treatments, and to allow respect of patients’ choices regarding their plan and place of care. Early palliative care has effectively been shown to be a new paradigm of oncologic treatment and palliative care, improving QOL, depression, prognostic understanding, and health service use in patients with advanced cancer [34].

PCPs also reported having experienced the specificities of interprofessional teams in palliative care. The interprofessional nature of palliative care teamwork with a view to whole person care has been described before. This teamwork differs from traditional models where the team is led by physicians, which are primarily focused on a disease process [35].

Regarding the advance directives and advance care planning, PCPs reflected how the study made them conscious of their role in actively helping patients define their preferences. The study led them to shift from the “after you” position in which PCPs expect patients to address the subject, while patients expect PCPs to address it first [28].

PCPs expressed how these rich discussions with their patients helped them to know them better, empowering them to become the patient’s advocate while facing specialists or hospital caregivers. Again, it underlined the new experienced paradigm for participating PCPs, shifting from helping patients to better die to a new paradigm in which they improve the quality of end-of-life.

When coming to the analysis of the difficult patient recruitment with the participating PCPs, it showed that addressing end-of-life discussions remained difficult. The simple words “palliative care” were difficult to express to patients and some participants suggested to rename palliative care as “patient support” or “support treatment” so as to smoothen the discussion; as previously described [25]. Similarly, as described in the literature, the participating PCPs expressed their fear of stealing patients’ hope (regarding their treatment and life expectancy) when trying to address end-of-life issues. It made them feel uncomfortable in the role of “announcer of bad news” [28]. The fear of adding the load for their already frail and very sick patients of confronting a stranger asking a range of questions also contributed to caution while recruiting patients.

### Suggestions for future research

As suggested by participating PCPs, further research could concentrate on the PCPs attitudes and change of roles as indicators for the impact of the intervention, as well as the completion of advance directives or advance care planning. This would encourage more training of PCPs and allow further action research that could facilitate early palliative care and PCPs involvement for advanced cancer patients.

### Strengths and limitations

Strengths of our study include the qualitative approach of PCPs feedback that gives a very rich view of their thoughts, attitudes and roles. In addition to the low participation rate, another limitation is the possible selection of PCPs interested in palliative care, that may limit the generalisability of the intervention’s impact.

## Conclusions

This pilot study showed that such recruitment would not be feasible for a follow up RCT using the current design as too few patients were recruited, though the acceptability of the intervention was excellent and shows that PCPs benefit from interventions helping them to overcome barriers to address end-of-life issues, advance directives and advance care planning. It enhances interprofessional teamwork, palliative care and respect of patient preferences of care. PCPs described that it changed their paradigm of palliative care, investing their role to facilitate patients’ “better life for end-of-life instead of better dying”.

Future studies focusing on their changes of attitude and completion of advance directives and advance care planning as well as collaboration in interprofessional teamwork should contribute to the dissemination of better care for advanced cancer patients.

## Data Availability

The datasets used and/or analysed during the current study are available from the corresponding author on reasonable request.

## Abbreviation

PCP: Primary care physician

## References

[1] Office fédéral de la statistique OFS. Cancer-statistiques 2017 Neuchâtel 2020,12. Available from: https://www.bfs.admin.ch/bfs/fr/home/statistiques/sante/etat-sante/maladies/cancer.html. Accessed 26 June 2021.

[2] Anvik T, Holtedahl KA, Mikalsen H (2006). “When patients have cancer, they stop seeing me”--the role of the general practitioner in early follow-up of patients with cancer--a qualitative study. BMC Fam Pract.

[3] Aabom B, Kragstrup J, Vondeling H, Bakketeig LS, Stovring H (2006). Does persistent involvement by the GP improve palliative care at home for end-stage cancer patients?. Palliat Med.

[4] Temel JS, Greer JA, Muzikansky A, Gallagher ER, Admane S, Jackson VA, Dahlin CM, Blinderman CD, Jacobsen J, Pirl WF, Billings JA, Lynch TJ (2010). Early palliative care for patients with metastatic non-small-cell lung cancer. N Engl J Med.

[5] Aabom B, Pfeiffer P (2009). Why are some patients in treatment for advanced cancer reluctant to consult their GP?. Scand J Prim Health Care.

[6] Centre NBC (2003). Clinical practice guidelines for the psychosocial care of adults with cancer.

[7] Meiklejohn JA, Mimery A, Martin JH, Bailie R, Garvey G, Walpole ET, Adams J, Williamson D, Valery PC (2016). The role of the GP in follow-up cancer care: a systematic literature review. J Cancer Surviv.

[8] Puchalski CM (2012). Spirituality in the cancer trajectory. Ann Oncol.

[9] Balboni TA, Vanderwerker LC, Block SD, Paulk ME, Lathan CS, Peteet JR, Prigerson HG (2007). Religiousness and spiritual support among advanced cancer patients and associations with end-of-life treatment preferences and quality of life. J Clin Oncol.

[10] Hamilton IJ, Morrison J, Macdonald S (2017). Should GPs provide spiritual care?. Br J Gen Pract.

[11] Slort W, Schweitzer BP, Blankenstein AH, Abarshi EA, Riphagen II, Echteld MA, Aaronson NK, der Horst HEV, Deliens L (2011). Perceived barriers and facilitators for general practitioner-patient communication in palliative care: a systematic review. Palliat Med.

[12] Otte IC, Jung C, Elger BS, Bally K (2014). Advance directives and the impact of timing. A qualitative study with Swiss general practitioners. Swiss Med Wkly.

[13] Selman LE, Brighton LJ, Robinson V, George R, Khan SA, Burman R, Koffman J (2017). Primary care physicians’ educational needs and learning preferences in end of life care: a focus group study in the UK. BMC Palliat Care.

[14] Russel B, Ward A (2011). Deciding what information is necessary: do patients with advanced cancer want to know all the details?. Cancer Manag Res.

[15] Barclay S, Maher J (2010). Having the difficult conversations about the end of life. BMJ..

[16] Brighton LJ, Bristowe K (2016). Communication in palliative care: talking about the end of life, before the end of life. Postgrad Med J.

[17] Vermandere M, De Lepeleire J, Smeets L, Hannes K, Van Mechelen W, Warmenhoven F (2011). Spirituality in general practice: a qualitative evidence synthesis. Br J Gen Pract.

[18] Barclay S, Wyatt P, Shore S, Finlay I, Grande G, Todd C (2003). Caring for the dying: how well prepared are general practitioners? A questionnaire study in Wales. Palliat Med.

[19] Boyd K, Mason B, Kendall M, Barclay S, Chinn D, Thomas K, Sheikh A, Murray SA (2010). Advance care planning for cancer patients in primary care: a feasibility study. Br J Gen Pract.

[20] Bruera E, Kuehn N, Miller MJ, Selmser P, Macmillan K (1991). The Edmonton symptom assessment system (ESAS): a simple method for the assessment of palliative care patients. J Palliat Care.

[21] Ware J, Kosinski M, Keller SD (1996). A 12-item short-form health survey: construction of scales and preliminary tests of reliability and validity. Med Care.

[22] Zigmond AS, Snaith RP (1983). The hospital anxiety and depression scale. Acta Psychiatr Scand.

[23] Peterman AH, Fitchett G, Brady MJ, Hernandez L, Cella D (2002). Measuring spiritual well-being in people with cancer: the functional assessment of chronic illness therapy--spiritual well-being scale (FACIT-Sp). Ann Behav Med.

[24] Asch S, Connor SE, Hamilton EG, Fox SA (2000). Problems in recruiting community-based physicians for health services research. J Gen Intern Med.

[25] Thomas HR, Deckx L, Sieben NA, Foster MM, Mitchell GK (2020). General practitioners’ considerations when deciding whether to initiate end-of-life conversations: a qualitative study. Fam Pract.

[26] Deckx L, Thomas HR, Sieben NA, Foster MM, Mitchell GK (2020). General practitioners’ practical approach to initiating end-of-life conversations: a qualitative study. Fam Pract.

[27] Szekendi MK, Vaughn J, McLaughlin B, Mulvenon C, Porter-Williamson K, Sydenstricker C, Williamson M (2018). Integrating palliative care to promote earlier conversations and to increase the skill and comfort of nonpalliative care clinicians: lessons learned from an interventional field trial. Am J Hosp Palliat Care.

[28] Almack K, Cox K, Moghaddam N, Pollock K, Seymour J (2012). After you: conversations between patients and healthcare professionals in planning for end of life care. BMC Palliat Care..

[29] Trankle SA, Shanmugam S, Lewis E, Nicholson M, Hillman K, Cardona M (2020). Are we making Progress on communication with people who are near the end of life in the Australian health system? A Thematic Analysis. Health Commun.

[30] Momen NC, Barclay SI (2011). Addressing ‘the elephant on the table’: barriers to end of life care conversations in heart failure - a literature review and narrative synthesis. Curr Opin Support Palliat Care.

[31] Pocock LV, Wye L, French LRM, Purdy S (2019). Barriers to GPs identifying patients at the end-of-life and discussions about their care: a qualitative study. Fam Pract.

[32] Mason B, Nanton V, Epiphaniou E, Murray SA, Donaldson A, Shipman C, Daveson BA, Harding R, Higginson IJ, Munday D, Barclay S, Dale J, Kendall M, Worth A, Boyd K (2016). ‘My body’s falling apart.’ Understanding the experiences of patients with advanced multimorbidity to improve care: serial interviews with patients and carers. BMJ Support Palliat Care.

[33] Murray SA, Boyd K, Sheikh A (2005). Palliative care in chronic illness. BMJ..

[34] Bauman J, Temel JS (2014). The integration of early palliative care with oncology care: the time has come for a new tradition. J Natl Compr Cancer Netw.

[35] Blacker S, Deveau C (2010). Social work and interprofessional collaboration in palliative care. Progress Palliat Care.

